# Plasma Imidazole Propionate Is Positively Correlated with Blood Pressure in Overweight and Obese Humans

**DOI:** 10.3390/nu13082706

**Published:** 2021-08-06

**Authors:** Jamie van Son, Mireille J. Serlie, Marcus Ståhlman, Fredrik Bäckhed, Max Nieuwdorp, Judith Aron-Wisnewsky

**Affiliations:** 1 Department of Vascular Medicine, Amsterdam University Medical Centers, Location AMC, Meibergdreef 9, 1105 AZ Amsterdam, The Netherlands; j.vanson@amsterdamumc.nl (J.v.S.); m.nieuwdorp@amsterdamumc.nl (M.N.); 2 Department of Endocrinology and Metabolism, Amsterdam University Medical Centers, Location AMC, Meibergdreef 9, 1105 AZ Amsterdam, The Netherlands; m.j.serlie@amsterdamumc.nl; 3 Wallenberg Laboratory, University of Gothenburg, Bruna Stråket 16, SE-413 45 Gothenburg, Sweden; marcus.henricsson@wlab.gu.se (M.S.); Fredrik.Backhed@wlab.gu.se (F.B.); 4 Department of Clinical Physiology, Sahlgrenska University Hospital, Region Västra Götaland, Blå Stråket 5, SE-413 45 Gothenburg, Sweden; 5 Novo Nordisk Foundation Center for Basic Metabolic Research, Faculty of Health Sciences, University of Copenhagen, Blegdamsvej 3B, 2200 Copenhagen, Denmark; 6 Department of Internal Medicine, Amsterdam University Medical Centers, Location AMC, Meibergdreef 9, 1105 AZ Amsterdam, The Netherlands; 7 Nutrition and Obesities, Systemic Approaches (NutriOmics) Research Group, Sorbonne Université, Inserm, 75013 Paris, France; 8 Assistante Publique Hôpitaux de Paris, Nutrition Department, Pitié-Salpêtrière Hospital, CRNH Ile de France, 75013 Paris, France

**Keywords:** imidazole propionate, cardiovascular disease, insulin resistance, obesity, gut microbiota, hyperinsulinemic-euglycemic clamp

## Abstract

Background: The gut microbiota and its metabolites are essential for host health and dysbiosis has been involved in several pathologic conditions such as type 2 diabetes (T2D) and cardiovascular disease (CVD). Recent studies have identified that plasma imidazole propionate (ImP), a microbial-produced metabolite, is increased in patients with prediabetes and T2D. More recently, ImP was found to be significantly increased in patients with overt CVD. Here, we aimed to investigate the association between ImP and CVD risk factors: blood pressure, HDL-cholesterol, LDL-cholesterol and insulin-resistance in overweight and obese subjects without T2D or use of any metabolic diseases-related medication. Methods: Plasma metabolites, including ImP, were determined in 107 male or post-menopausal women with overweight/obesity, but without T2D. Insulin-sensitivity was assessed with the gold standard method: the hyperinsulinemic-euglycemic clamp using the isotope [6,6-^2^H_2_] glucose and expressed as glucose rate of disposal (Rd) for peripheral insulin sensitivity and suppression of endogenous glucose production (EGP) for hepatic insulin sensitivity. Results: Partial correlation analysis controlled for BMI and age showed a significant correlation between ImP and diastolic blood pressure (r_s_ = 0.285, *p* = 0.004) and a borderline significance with systolic blood pressure (r_s_ = 0.187, *p* = 0.060); however, systolic and diastolic blood pressure did not correlate with ImP precursor histidine (r_s_ = 0.063, *p* = 0.526 and r = −0.038, *p* = 0.712, respectively). We did not find a correlation between ImP with LDL-cholesterol or HDL-cholesterol (r_s_ = −0.181, *p* = 0.064 and r_s_ = 0.060, *p* = 0.546, respectively). Furthermore, there was no association between plasma ImP concentrations and Rd and EGP suppression. Conclusion: In this cohort with overweight/obese subjects without T2D, plasma ImP concentrations were positively correlated with diastolic blood pressure but not with insulin-sensitivity.

## 1. Introduction

Cardiovascular disease (CVD) is the global leading cause of death, with an estimated 31% of all deaths in 2016 [1]. There are many known risk factors, which include age, sex, smoking status, blood cholesterol, blood pressure, type 2 diabetes (T2D), body mass index (BMI) and central obesity, as well as socio-economic status and family history of premature CVD [2,3]. Obesity is a major cause for the increase in CVD as it is associated with other risk factors, such as abnormal lipids, hypertension and T2D [4]. T2D is a metabolic disorder characterized by elevated blood glucose levels due to insulin resistance and β-cell dysfunction, and increases the risk for morbidity and premature death [5,6]. However, insulin resistance in the absence of diabetes has also shown to be an independent risk factor for CVD [7,8].

The gut microbiota is essential for host health and is involved in several metabolic processes such as the degradation of dietary elements and production of microbial metabolites [9]. Importantly, microbial dysbiosis has been associated with several chronic diseases, such as obesity, T2D and CVD [10,11,12,13,14]. Furthermore, the gut microbiota displays altered composition and function during metabolic diseases, which translates into different circulating metabolite profiles [15]. A recently discovered metabolite: imidazole propionate (ImP), derived from microbial-metabolism of dietary histidine, has been found to be increased in the systemic circulation of patients with T2D. Murine studies have subsequently identified that ImP negatively affects glucose tolerance and impairs insulin signaling through the activation of a mechanistic target of rapamycin complex 1 (mTORC1) [16]. Recent findings also demonstrated a progressive increase of ImP from patients with normal glucose tolerance to prediabetes to overt T2D [17]. Additionally, circulating ImP concentrations were significantly increased in patients with CVD after adjustment for age, gender, BMI, ethnicity and diabetes status [17]. However, since this cohort already displayed overt CVD and were heavily treated for their CVD risk factor, they were not able to explore whether ImP was associated with known risk factors such as hypertension and cholesterol [18].

Here, we aimed to assess the association between circulating plasma ImP concentrations and risk factors for CVD, namely blood pressure, high-density lipoprotein (HDL) cholesterol and low-density lipoprotein (LDL) cholesterol, in overweight/obese humans without T2D and who were still naïve from any CVD medications. Secondly, we aimed to assess whether ImP was associated with peripheral and hepatic insulin resistance, assessed by the gold standard method, in obese humans without prediabetes or T2D.

## 2. Materials and Methods

### 2.1. Study Participants

We included adult subjects who had participated in five previous conducted studies at the Academic Medical Center (Amsterdam, Netherlands) between May 2011 and March 2017 [19,20,21,22,23]. Inclusion criteria were: BMI > 25kg/m^2^, men or postmenopausal women and availability of data on blood pressure, fasting blood samples and insulin sensitivity assessed with the two-step hyperinsulinemic euglycemic clamp. Exclusion criteria included: age > 71 years and the use of any medication, including proton pump inhibitors and antibiotics in the last 3 months. Other exclusion criteria were consumption of more than 14 units of alcohol a week, smoking, a history of cholecystectomy or any previous cardiovascular event. No subjects had overt T2D defined as 2 fasting plasma glucose ≥ 7 mmol/L and/or Hba1c ≥ 6.5% (48 mmol/mol) and/or an abnormal oral glucose tolerance test [24]. All procedures were approved by the Academic Medical Center medical ethics committee and all subjects provided written informed consent in accordance with the Declaration of Helsinki.

### 2.2. Two-Step Hyperinsulinemic Euglycemic Clamp

Insulin sensitivity was assessed with the two-step hyperinsulinemic euglycemic clamp and use of the [6,6-^2^H_2_]glucose tracer (>99% enriched; Cambridge Isotopes, Andover, MA, USA). After a 12 h overnight fast, subjects were admitted to the research unit and two peripheral venous catheters were placed. Fasting blood samples were collected and macronutrient dietary intake was monitored with a food diary in the preceding week. One catheter was used for infusion of [6,6-^2^H_2_] glucose, 20% glucose enriched with 1% [6,6-^2^ H_2_]glucose and insulin (Actrapid; Novo Nordisk Farma, Alphen aan de Rijn, Netherlands). The other catheter was arterialized with a heated hand box (60 °C) and used for blood sampling. At t = −2 h, infusion was started with [6,6-^2^H_2_]glucose (prime 11 μmol kg^−1^; continuous infusion 0.11 μmol kg^−1^ min^−1^) and continued until the end of the experiment. After 2 h (t = 0 h), infusion of insulin was started. During step one, insulin was infused at a rate of 20 mU·m^−2^·min^−1^ and increased during step two to 60 mU·m^−2^·min^−1^. Plasma glucose concentrations were measured every 5–10 min and stabilized at 5.0 mmol/L with variable infusion rates of 20% glucose enriched with 1% [6,6-^2^H_2_]glucose. Blood samples were collected with a 5 min interval to assess glucose enrichments and glucoregulatory hormones at t = 0 h, t = 2 h (the end of step one) and t = 4 h (the end of step two). Rates of disposal (Rd) of glucose and endogenous glucose production (EGP) were calculated using the modified versions of the Steele equations [25]. Peripheral insulin sensitivity and hepatic insulin sensitivity are expressed by the insulin-mediated Rd assessed at t = 4 h and suppression of EGP at t = 2 h relative to basal, respectively.

### 2.3. Measurement of Metabolites

Untargeted semi-quantitative plasma metabolomics of the fasting blood samples was performed by Metabolon (Durham, NC, USA), using ultra-high performance liquid chromatography coupled to tandem mass spectrometry, which was confirmed using targeted measurement of plasma ImP and urocanate [16].

### 2.4. Statistical Analysis

Categorical variables are presented as count and percentage (%). Continuous variables are presented as mean ± standard deviation (SD) and median [interquartile range [IQR]] for parametric and nonparametric data, respectively. Groups were compared with an unpaired *t*-test or the Mann–Whitney U test as appropriate. Pearson or Spearman rank correlation were used to test correlations between parametric or non-parametric data, respectively. Analyses were adjusted for the confounders BMI and age, as they have been associated with either plasma metabolites or with cardiovascular risk factors such as insulin resistance, blood pressure and cholesterol. Results with a *p*-value < 0.05 were considered statistically significant. Analyses were performed using IBM SPSS statistics version 26 (SPSS, Inc., Chicago, IL, USA).

## 3. Results

We included 107 subjects with a median age of 56 (51–63) years, BMI 33.6 (31–36) kg/m^2^ and 89.7% were male. With use of the validated cut-off Rd < 37.3 μmol·kg^−1^min^−1^ for peripheral insulin resistance, we categorized our cohort into insulin resistant subjects *n* = 76 (71%) and insulin sensitive subjects *n* = 31 (29%) [26]. Between these two groups, there was no significant difference in age, sex or BMI. Additionally, there was no difference in blood pressure, HDL-cholesterol or LDL-cholesterol between insulin-sensitive and insulin-resistant subjects. While insulin-resistant patients had an increased total calorie intake, their protein intake did not differ, Table 1. Although fiber intake was increased, after correction for total intake, there was no difference between the groups (*p* = 0.132).

First, analyses of plasma metabolites showed that semi-quantitative metabolomics measurement of ImP (nM) and urocanate (nM) highly correlated with their quantitative measures (r = 0.912, *p* < 0.001 and r = 0.4, *p* < 0.001, respectively).

**Figure 1:**
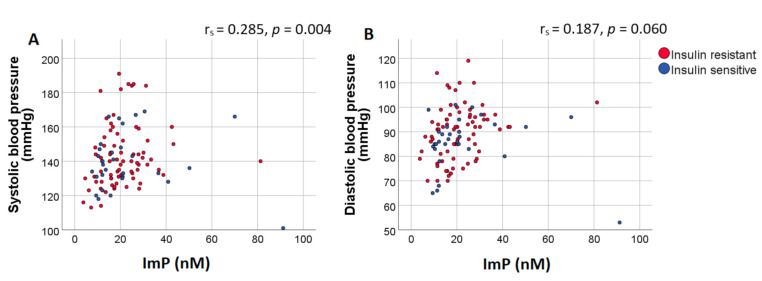
Plasma ImP concentrations positively correlate with diastolic blood pressure. Scatter plot of plasma ImP concentrations (nM) and (**A**) systolic and (**B**) diastolic blood pressure. Results of the Spearman correlation indicated that there was a significant positive association between ImP and diastolic blood pressure. Spearman correlation coefficients and *p*-values were calculated using partial correlations adjusted for BMI and age. Red dots represent insulin resistant subjects (Rd < 37.3 μmol·kg^−1^min^−1^) while blue dots represent insulin sensitive subjects (Rd > 37.3 μmol·kg^−1^min^−1^). Abbreviations: ImP, imidazole propionate.

Next, we analyzed the correlation of ImP with blood pressure, HDL-cholesterol and LDL-cholesterol, controlled for BMI and age. Diastolic blood pressure positively correlated with ImP (r_s_ = 0.285, *p* = 0.004)). Correlation between systolic blood pressure and ImP approached significance after controlling for BMI and age (r_s_ = 0.187, *p* = 0.060), Figure 1. Conversely, histidine did not correlate with systolic or diastolic blood pressure (r_s_ = 0.063, *p* = 0.526 and r = −0.038, *p* = 0.712, respectively). We did not find a correlation between ImP with LDL-cholesterol (r_s_ = −0.181, *p* = 0.064) or between ImP and HDL-cholesterol (r_s_ = 0.060, *p* = 0.546), which was additionally adjusted for sex.

**Figure 2:**
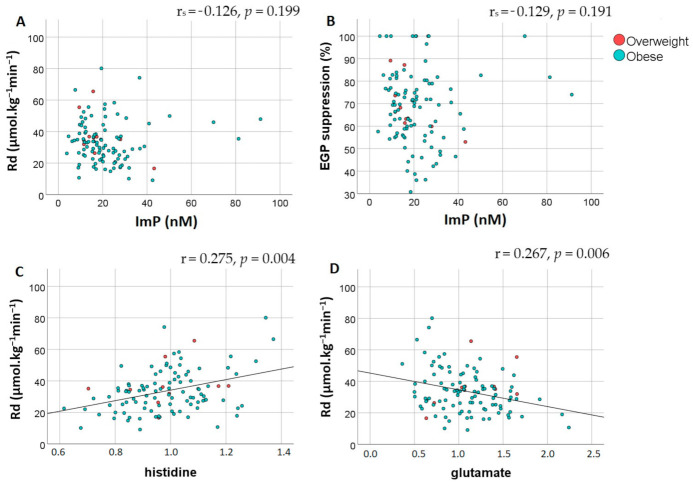
Peripheral and hepatic insulin sensitivity are not associated with imidazole propionate but do correlate with histidine and glutamate. Spearman partial correlation analysis adjusted for BMI and age showed an absence of correlation between plasma imidazole propionate concentrations (nM) and (**A**) peripheral insulin resistance assessed by Rd (μmol·kg^−1^min^−1^) and (**B**) hepatic insulin resistance assessed by EGP suppression (%). Pearson correlation indicated that there was a significant correlation between Rd and (**C**) plasma histidine levels and (**D**) plasma glutamate levels. Red dots represent overweight subjects while blue dots represent subjects with obesity. Abbreviations: Rd, rate of disposal; EGP, endogenous glucose production; ImP, imidazole propionate.

Lastly, we assessed the association between plasma ImP levels and insulin resistance. There was no significant difference in plasma ImP concentrations between insulin sensitive and insulin resistant subjects (16.3 nM (11.4–25.2) vs. 19 nM (13.8–26.7), respectively; *p* = 0.426). Moreover, correlation analyses adjusted for BMI and age confirmed the lack of association between Imp with Rd and EGP suppression, Table 2, Figure 2A,B. Additional analyses of other metabolites in the histidine degradation pathway did not show a correlation between Rd and urocanate (r = 0.141, *p* = 0.148). However, histidine and glutamate levels did significantly correlate with Rd, Figure 2C,D. Importantly, there was no correlation between histidine and ImP (r = −0.121, *p* = 0.215).

## 4. Discussion

Overall, the data from this cohort of overweight/obese subjects without T2D and naïve from any CVD medication show a correlation between plasma ImP concentrations and diastolic blood pressure, but not with insulin resistance. As previously described, a recent study found that ImP was indeed associated with CVD. Hypertension is one of the most important risk factors for CVD [27], and diastolic hypertension has been shown to independently increase the risk of cardiovascular events [28], so one could hypothesise that a negative effect of ImP on blood pressure could contribute to the development of CVD. Conversely, a study in rats reported that oral supplementation of histidine, the precursor to ImP, was protective against high-salt diet induced hypertension [29], yet this study neither assessed ImP concentration nor gut microbiota dysbiosis. Importantly, circulating ImP concentrations are not impacted by histidine intake within the usual diet, suggesting that increased ImP levels observed in T2D individuals do not originate from increased histidine intake [17]. Additional analysis in our cohort did not find an association with two other important risk factors, namely LDL-cholesterol and HDL-cholesterol [30].

We did not find a significant association between plasma ImP concentrations and peripheral or hepatic insulin resistance. Our data may seem contrary to previous reports; however, it is important to note that our cohort consists of subjects without type 2 diabetes in a very homogeneous range of BMI without any medication or any overt chronic diseases except for metabolic syndrome. Furthermore, the range of ImP values is much below those levels published in studies which included patients from a wide range of BMI and metabolic diseases’ severity [16,17]. As discussed previously, insulin resistance is a risk factor for CVD, and several studies have found an association between insulin resistance and hypertension [31,32]. In our cohort, however, we did not find a difference in blood pressure, HDL-cholesterol or LDL-cholesterol between insulin-resistant and insulin-sensitive subjects. This could imply that, if ImP does affect blood pressure, it would more likely be through other mechanisms than insulin resistance. This could possibly be a direct effect or through other intermediate pathways, such as the effect of ImP on systemic inflammation or mTOR activation [17,33,34].

The strength of this study lays in the assessment of insulin resistance by the gold standard stable isotope clamp method as well as the inclusion of treatment naïve subjects without overt diseases. However, we acknowledge several limitations of our study. Literature has shown that gut microbiota composition and function are subjected to progressive changes from normoglycemic state, to prediabetes to overt T2D, and we herein did not perform gut microbiota analysis. Furthermore, it is important to note that our cohort consists of a homogenous group of middle-aged overweight/obese subjects, who are mostly male. This could be the reason why, in our hand, ImP values do not display the large range of systemic values and inter-individual variability previously observed [35].

## 5. Conclusions

In summary, in this cohort with overweight/obese subjects without T2D and a varying degree of insulin-resistance, plasma ImP concentrations were positively correlated with diastolic blood pressure but not with insulin-sensitivity. The role ImP has in insulin resistance and related comorbidities is complex and our data combined with previous reports suggest that this role could progress as T2D develops. Future research including larger groups with a wider range of metabolic parameters could further unravel where ImP stands in the pathogenesis of cardiovascular disease and insulin resistance. Thus, future studies are needed to investigate if there is a causal link between ImP and CVD and which underlying mechanisms could be involved.

## Figures and Tables

**Table 1:** Characteristics of study participants.

	Insulin Resistant (*n* = 76)	Insulin Sensitive (*n* = 31)	*p*-Value
**Demographic characteristics**			
Male sex (%)	69 (91)	27 (87)	0.826
Age (years)	56 (9)	56 (7)	0.758
BMI (kg/m^2^)	33.9 (31.1–37.6)	33.0 (30.6–35.0)	0.206
**Metabolic characteristics**			
Systolic blood pressure (mmHg)	140 (130–152)	137 (131–148)	0.400
Diastolic blood pressure (mmHg)	90 (11)	86 (11)	0.092
Fasting plasma glucose (mmol/L)	5.9 (0.7)	5.7 (0.6)	0.136
Fasting insulin (pmol/L)	122 (89–149)	69 (60–96)	<0.001
HbA1c (mmol/mol)	39 (4)	38 (4)	0.226
Total cholesterol (mmol/L)	5.4 (4.8–6.2)	4.9 (4.6–6.4)	0.421
Low-density lipoprotein (mmol/L)	3.5 (2.9–4.3)	3.4 (2.6–4.2)	0.291
High-density lipoprotein (mmol/L)	1.1 (0.9–1.3)	1.2 (1.0–1.5)	0.115
Triglycerides (mmol/L)	1.5 (1.2–1.8)	1.3 (1.1–1.7)	0.282
Aspartate aminotransferase (U/L)	25 (21–29)	23 (22–26)	0.264
Alanine Aminotransferase (U/L)	33 (26–41)	27 (21–34)	0.018
C-reactive protein (mg/L)	2.1 (1.4–4.2)	1.7 (1.0–4.0)	0.760
**Dietary intake**			
Total calorie (kcal/day)	2010 (461)	1756 (341)	0.011
Fat (gram/day)	75 (20)	65 (25)	0.076
Carbohydrate (gram/day)	202 (166–239)	170 (141–222)	0.087
Protein (gram/day)	87 (19)	85 (17)	0.669
Fiber (gram/day)	19 (17–21)	15 (13–19)	0.009
**Hyperinsulinemic euglycemic clamp**			
Rd (µmol.kg^−1^min^−1^)	27.5 (20.8–33.6)	48.6 (43.9–55.4)	<0.001
EGP suppression (%)	63.7 (14.5)	81.5 (16.2)	<0.001

Categorical data are count (%) and compared by chi-square test. Continuous data are median (IQR] or mean (standard deviation) and compared by Mann–Whitney U test or *t*-test, respectively. Subjects are categorized as insulin resistant (Rd < 37.3 μmol·kg^−1^min^−1^) or insulin sensitive (Rd > 37.3 μmol·kg^−1^min^−1^). Abbreviations: BMI, body mass index; HbA1c, Hemoglobin A1c; Rd, rate of disposal; EGP, endogenous glucose production.

**Table 2:** Correlation imidazole propionate (nM) with cardiovascular risk factors and insulin resistance.

	r_s_	*p*-Value
Systolic blood pressure (mmHg)	0.187	0.060
Diastolic blood pressure (mmHg)	0.285	0.004
Low-density lipoprotein (mmol/L)	−0.181	0.064
High-density lipoprotein (mmol/L)	0.060	0.546
Rd (µmol·kg^−1^min^−1^)	−0.126	0.199
EGP suppression (%)	−0.129	0.191

Spearman Rank correlation analysis of plasma ImP (nM) concentrations controlled for BMI and age. Abbreviations: Rd, rate of disposal; EGP, endogenous glucose production.

## Data Availability

The data presented in this study are available on request from the corresponding author.
